# Proximal Femoral Allograft for Major Segmental Femoral Bone Loss: A Systematic Literature Review

**DOI:** 10.4061/2011/257572

**Published:** 2011-10-13

**Authors:** B. A. Rogers, A. Sternheim, D. Backstein, O. Safir, A. E. Gross

**Affiliations:** Department of Orthopaedic Surgery, Mount Sinai Hospital, Toronto, ON, Canada M5G 1X5

## Abstract

As the indications for total hip arthroplasty increase, the prevalence of extensive proximal femoral bone loss will increase as a consequence of massive osteolysis, stress shielding and multiple revisions. Proximal femoral bone stock deficiency provides a major challenge for revision hip arthroplasty and is likely to account for a significant future caseload. Various surgical techniques have been advocated included impaction allografting, distal press-fit fixation and massive endoprosthetic reconstruction. This review article provides a systematic review of the current literature to assess the outcome of revision hip arthroplasty using allograft to reconstruction massive proximal femoral bone loss.

## 1. Introduction

As the need for total hip arthroplasty increases, the incidence of extensive proximal femoral bone loss will increase as a consequence of massive osteolysis, stress shielding and multiple revisions [1–5]. Proximal femoral bone stock deficiency provides a major challenge for revision hip arthroplasty and is likely to account for a significant future caseload [6]. 

Various surgical techniques have been advocated included impaction allografting techniques [7, 8], distal press-fit fixation [9, 10], and massive endoprosthetic reconstruction [11–13]. Individual studies have reported a 58% to 84% survivorship of massive endoprosthetic reconstruction (or megaprostheses) with average followup ranging from 5 to 10 years [11–13]. A recent retrospective review of 403 proximal femoral replacements (endoprosthetic reconstructions) from five institutions reported a 10- and 15-year survival rate of 75%, with mechanical causes being the commonest mode of failure [14]. 

A proximal femoral allograft reconstruction requires the use of a prosthesis bridging the host-allograft junction and obtaining fixation in the distal femur. The enhancement of future bone stock is an important advantage purported to this method of reconstruction that has been utilized in proximal femoral bone loss secondary to tumors and aseptic osteolysis. Differences in the morphology of the host-allograft junction, the use of cement, and the method of attachment of the host abductor musculature have all been described.

The three principal aims of this systematic review were as follows:

1. to document variations in the surgical techniques used,
2. to assess the clinical outcome of allograft prosthesis composites (APC) for massive proximal femoral bone loss,
3. to quantify complication rates in relation to the surgical technique used.

## 2. Methods

A comprehensive search of the MEDLINE, EMBASE, and the National Institutes of Health online database PubMed from the earliest records to the time of review (January 2011) was performed. The following Medical Subject Headings (MeSH) terms were used: “allograft,” “composite graft” in the manuscript title, and “proximal femoral” in the manuscript abstract. The keywords were used as both text words and Medical Search Headings (MeSH terms).

Two authors (B. A. Rogers, A. Sternheim) independently applied the search strategy to the different databases and reviewed the selected references. Titles, abstracts and papers were reviewed independently.

The following inclusion criteria were used:

1. studies retrieved by the database search using the Medical Subject Headings detailed above,
2. studies specifically reporting outcomes relating to proximal femur composite.

The following exclusion criteria were used:

1. non-English language,
2. case reports,
3. review articles,
4. not relating to human surgery,
5. patients with advanced oncological pathology,
6. followup less than 2 years.

Where more than one publication existed relating to the outcomes of same cohort of patients from the same institution, the most recent publication only was used.

Full-text manuscripts were obtained and reviewed for the studies identified using the above criteria. The method of review followed the authoritative methodology described by Mohit [15].

**Figure 1:**
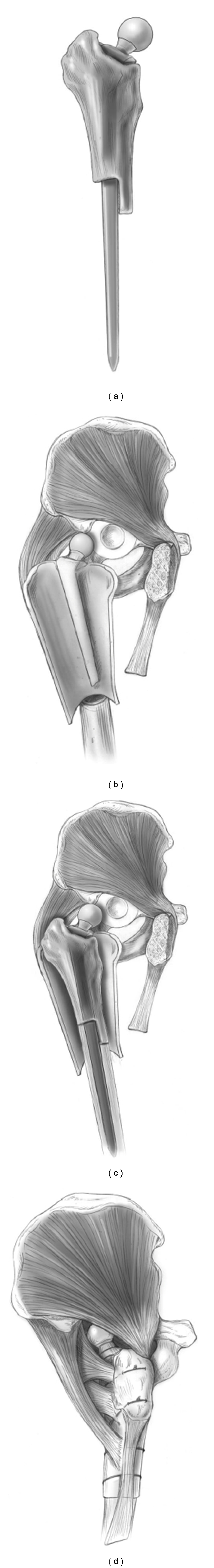
(a) Prosthesis cemented into allograft. (b) Trochanteric slide approach to hip, with lateral cortex osteotomy to facilitate removal of in situ femoral component. (c) Allograft-prosthesis composite inserted into host, with junctional step cut. (d) Remnants of host proximal femur are fixed around allograft, especially at the allograft-host junction, and the greater trochanter reattached.

**Figure 2:**
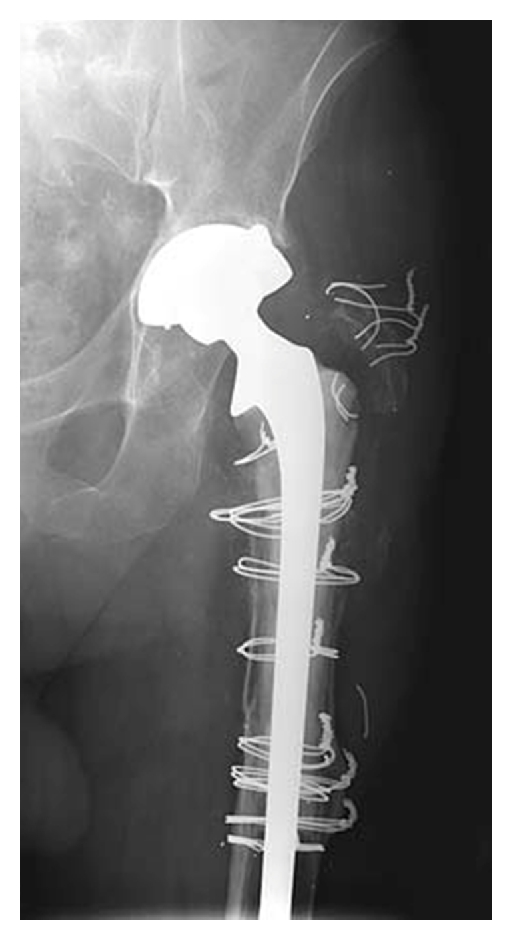
A radiograph 17 years after proximal femoral allograft (reprinted from Safir et al. [19]).

Allograft-prosthetic composite (APC) is a technique used to restore bone stock and mechanical stability to the proximal femur (see Figures 1(a)–1(d), and 2). The studies analyzed in this literature review consider a single technique, APC, rather than a single diagnosis; this technique has been utilized for oncological and nononcological surgery.

Eight studies report on APC used in non-oncological conditions (septic or aseptic loosening) and six report on surgeries performed for malignant or nonmalignant proximal femoral pathology. Two studies report on patient cohorts with both indications.

The primary outcome of interest was further revision of the femoral component and the secondary outcomes of interest were other complications such as infection, dislocation, and nonunion. 

Statistical analysis was performed on the selected papers to assess the pooled success rate. The effect (proportion) was calculated for every individual study and the pooled effect considering all the studies. 

## 3. Results

### 3.1. Studies

Sixteen studies reported on outcomes of proximal femoral composite allograft used to reconstruct major bone defects (see Table 1). All studies were retrospective case series and provide level IV evidence. All studies were published within the last fifteen years. Average followup ranged from 2 to 16.2 years. The total number of allograft reconstructions reported in all the studies was 498. The surgical techniques, clinical outcomes and complications were collated for all these published studies.

### 3.2. Surgical Techniques

The described surgical techniques varied, as shown in Tables 2 and 3. 

Four studies described the complete resection of the proximal femur as the approach employed; however, the transtrochanteric approach was the most common reported.

Regarding the morphology of the osteotomy used at the junction between the proximal allograft and distal host femur, 8 studies reported a transverse femoral osteotomy, 3 that were augmented with plate fixation to enhance stability at the allograft-host junction. The remainder of the studies reported either a step or oblique femoral osteotomy.

The management of the proximal host femur varied. In 9 studies the proximal host femur was fully resected, with 5 studies using the split host proximal femur as an onlay graft after the APC had been inserted. Two studies did not detail this aspect of the surgical technique. Four studies reported the use of cortical strut allografts to reinforce the allograft-host junction [6, 18, 22, 29], with one study reporting use in every case [22]. 

The techniques used for fixation of the prosthesis to the allograft, and for distal fixation to the host femur is shown in Table 3. There are 14 studies reporting cemented fixation of the prosthesis to the allograft; however, distal fixation varied with 6 uncemented, 4 cemented, 5 studies employed a variety of techniques and one study did not report. 

### 3.3. Clinical Outcomes

The primary outcome of interest was further revision of the femoral component, and Table 4 shows the reported failure rate and success rate for the allograft prosthesis composite in each study. The success rate was defined as the reported survivorship of the APC.

The total cohort included 498 patients with a mean follow up of 8.1 years (range 2 to 16.2 years). The pooled success rate was 81% (95% CI 77%–86%). 

However, the number of cases and length of followup varied substantially between the studies. For example, Roque et al. reported an 82% survivorship rate for 73 allograft prosthesis reconstructions at 6.7 years followup [23], whereas Safir et al. reported 15 year Kaplan-Meier survivorship data on 50 patients of 82% [19]. 

### 3.4. Complications

Table 4 details the reported major complications. The infection rate ranged from 0% to over 21%, with a pooled mean of 8%. The two studies with a reported infection rate of over 20% had only 14 and 15 patients, respectively [13, 29]. Conversely, the four studies reporting the lowest infection rates (0 to 4%) had a mean patient cohort of 40 patients [6, 16, 17, 20].

Dislocation is a significant postoperative complication, however five out of the sixteen studies did not report the incidence of dislocation [13, 24, 25, 27, 28]. For the eleven studies that did report dislocation rate the mean was 12.8% with a range 0% [16, 23] to 40% [18]. The mean reported dislocation rate in studies that used a technique of splitting the host proximal femur to use as an onlay graft was 9.8%, compared to 14.9% in studies that resected the entire proximal femur.

Failure of the APC, either resulting from aseptic loosening or fracture (Table 4) ranges from 0% to 28%. The mean reported aseptic loosening or fracture rate was 13.7% for studies that used cement for fixation into distal host femur, compared to 9.1% for those studies using uncemented fixation in the distal host femur. However, the difference was not statistically different.

## 4. Discussion

### 4.1. Clinical Outcome

Severe proximal femoral bone loss is creating an increasing caseload of complex cases for the reconstructive hip surgeon [6]. The use of allograft prosthesis composite (APC) is one surgical solution used to address this problem and restore mechanical stability to the proximal femur. This analysis reviews the surgical techniques, clinical outcomes and complication, incorporating a total patient cohort of 498 from sixteen studies with a mean follow up of 8.1 years (range 2 to 16.2 years). The pooled success rate was 81% (95% CI 77%–86%), see Table 4, and provides evidence that this technique is valid and durable when performed by suitable trained and experienced surgeons, in institutions with the facilities to support such complex surgery. 

### 4.2. Surgical Approaches and Complications

Surgical technique varied between the studies with regard to surgical approach, storage technique of the allograft bone, fixation techniques of the prosthesis to the proximal allograft, distal host femur and the junction between the allograft and host bone (see Table 2).

Several different surgical approaches were utilized in the reported studies. Four studies all pertain to tumour resection used a direct lateral approach with complete resection of the proximal femur. Trochanteric slide osteotomy was used in two studies both reported on patients who had revision of a failed hip arthroplasty. A transtrochanteric approach was reported by Vastel et al. and led to a high rate of trochanteric nonunion (25/34) with the authors recommending the use of a trochanteric plate to avoid proximal migration of the trochanter [20]. 

Trochanteric nonunion and abductor strength are also influenced by surgical approach. The trochanteric slide osteotomy aims to maintain the continuum of tissue from the abductors and the greater trochanter to the vastus lateralis. This approach has been reported to have a higher rate of trochanteric union [30]. The trochanteric slide osteotomy has been further modified to maintain the external rotators and thus improve hip stability [30–32]. 

The junctional osteotomy between the host femur and the proximal allograft was transverse, oblique or step-cut (see Table 2). A step-cut osteotomy may offer more rotational stability while an oblique osteotomy may offer more surface area for bone in-growth compared to a transverse osteotomy. Langlais et al. reported on two cases of loosening with junctional failure that they attributed to a lack of a step-cut osteotomy at the junction [16]. This junction may be further reinforced with strut allografts [6, 18, 22, 29]. Nonunion of the junction between the native femur and the proximal allograft causes macro motion at the junction that is treated with bone grafting, plating, and/or a strut allograft [16]. Host-allograft junctional nonunion may be reduced by augmentation with additional autologous bone graft and supporting it with either a plate or a strut allograft. Several studies highlight the bone union at the host-allograft junction as a key factor in achieving stability of the composite graft, and thereby lowering the chance of mechanical failure [17, 19, 21, 33].

Cement fixation of the prosthesis to the allograft with cementless fixation to the host femur was used in seven studies (see Table 3) [6, 19, 24, 25, 27–29]. The rationale for cement fixation in the allograft-prosthesis composite is that in-growth and on-growth would not be expected at the allograft prosthesis interface. Only Zmolek and Dorr reported a fully uncemented fixation of the prosthesis and allograft in 11 patients with similar rates of success compared to other studies [18]. 

Regarding distal fixation to the host femur, an uncemented technique was principally employed in nine studies [6, 18, 19, 22, 24, 25, 27–29], cemented in four studies [13, 16, 17, 20], mixed cemented and uncemented distal fixation in one study [21], and one study did not report whether or cement was used (see Table 3). 

For the studies that utilized cementless distal fixation, some employed a press-fit or interference technique whereas others used an oblique or step-cut junctional osteotomy. Safir et al. used an uncemented technique in the distal host femur with a step-cut or oblique osteotomy affording direct loading at the allograft-host femur junction [19]. The authors support the concept that direct loading of the host-allograft junction minimizes allograft resorption. The distal femur being initially reamed to the optimal size, with the proximal femoral allograft also reamed and broached until a good fit was achieved for the long-stem femoral prosthesis. The mismatch in the medullary sizes of the host bone and the allograft resulted in a good press-fit fixation never being achieved between the femoral stem and the distal host femur. Further, the allograft was never over reamed to accommodate a larger femoral component for the host femur. In contrast, Haddad et al. cemented the prosthesis to the distal femur thus stress shielding, the allograft and commented that this may explain the high rate of graft resorption (17%) observed [17].

Overall, cemented fixation in the distal host bone was associated with a higher rate of aseptic loosening or fracture (13.7%) when compared to uncemented distal fixation (9.1%; see Table 4). Whilst the difference was not statistically significant, the benefit of uncemented distal fixation is the reduced risk of junctional nonunion between the host femur and allograft.

The population cohorts, the duration, and complexity of the surgery result in infection rates for APC being greater than that for primary hip arthroplasty (see Table 4). Considering these factors, the pooled 8% infection rate is not unacceptable. The infection rate is; however, related to quantity performed with the lowest infection rates (0 to 4%) being reported in those studies with the greater number of cases [6, 16, 17, 20]. Although observer bias may influence this data, a greater caseload and experience is likely to be beneficial.

The use of native proximal femur with its soft-tissue attachments as an onlay graft around the composite allograft was reported in five studies [19–21, 27, 29]. This vascularised viable bone can promote in-growth into the allograft and preserves the abductor mechanism and short external rotators. These five studies report a lower mean dislocation rate of 9.8%, compared to 14.9% (see Table 4). From the surgical approaches detailed in these studies, the risk of dislocation may be minimized by:

1. preservation of the host posterior capsular structures if possible,
2. good biomechanical reconstruction of length, version and offset of the prosthesis-allograft construct,
3. maintaining the bone-soft tissue attachment to the host femur, to provide both mechanical stability and to act as a vascularised graft.

A constrained acetabular liner may be considered in cases of minimal abductor musculature.

## 5. Conclusion

The continued followup and analysis of this technique should be encouraged to refine and develop the management of massive proximal femoral bone loss. This review demonstrates that proximal femoral allografts for revision hip arthroplasty in femoral segmental bone loss do provide a durable solution, with current available evidence reporting a survivorship of 80%. Whilst a range of surgical techniques have been described, this study highlights the following:

1. high caseload is associated with a lower infection rate,
2. uncemented distal fixation is associated with a reduced the risk of aseptic loosening or fracture,
3. if available, using the host femur as an onlay graft enhances hip stability whilst acting as a vascularised graft.

## Figures and Tables

**Table 1:** Sixteen studies using allograft prosthetic composite in the treatment of proximal femoral bone loss, number of patients per study, primary diagnosis, and mean followup.

	Study	*n*	Primary diagnosis	Mean followup (yrs)
1	Chandler et al. [6]	30	Aseptic	2
2	Langlais et al. [16]	21	Tumor	6
3	Haddad et al. [17]	55	Tumor, Aseptic, Septic revision	8.8
4	Zehr et al. [13]	14	Tumor	10
5	Zmolek and Dorr [18]	15	Aseptic failure	2
6	Safir et al. [19]	50	Septic, aseptic	16.2
7	Vastel et al. [20]	44	Aseptic failure	7.1
8	Babis et al. [21]	72	Aseptic	12
9	Lee et al. [22]	15	Aseptic, septic loosening	4.2
10	Roque et al. [23]	73	Tumor	6.7
11	Biau et al. [24]	32	Tumor	5.6
12	Donati et al. [25]	22	Tumor	4.8
13	Farid et al. [26]	20	Tumor	6.3
14	Graham and Stockley [27]	25	Aseptic, septic loosening	4.5
15	Muscolo et al. [28]	37	Tumor	7.5
16	J. W. Wang and C. J. Wang [29]	15	Aseptic, septic loosening	7.6

**Table 2:** Surgical techniques used including approach, the type of femoral osteotomy performed at the host bone-allograft junction, and whether the host proximal femur was resected or split and used as an onlay graft. NR: not reported. Study numbers correlate with Table 1.

Study	*n*	Surgical approach	Femoral osteotomy	Host proximal femur
1	30	Trochanteric slide	Step cut (7) transverse (23)	NR
2	21	Complete resection	Step cut	Resected
3	55	NR	Transverse (28), step cut (12)	Resected
4	14	Complete resection	NR	Resected
5	15	Posterolateral (9), trochanteric Slide (2)	Oblique	Resected
6	50	Trochanteric slide	Step cut	Split and onlay
7	44	Transtrochanteric	Transverse	Split and onlay
8	72	Hardinge (44), posterior (11), transtrochanteric (17)	Step cut (62), telescoping (10)	Split and onlay
9	15	Transtrochanteric	Transverse (9), step cut (6)	NR
10	73	Complete resection	NR	Resected
11	32	Trochanteric slide (12), resection (20)	Transverse	Resected
12	22	Complete resection	Transverse	Resected
13	20	NR	NR	Resected
14	25	Trochanteric slide	Step cut	Split and onlay
15	37	Posterolateral (28), transtrochanteric (10)	Transverse	Resected
16	15	Transtrochanteric	Transverse	Split and onlay

**Table 3:** Table showing methods of implantation of prosthesis into allograft to form the allograft prosthesis composite (APC) and methods for securing APC to distal host femur. NR: not reported. Study numbers correlate with Table 1.

Study	Allograft-prosthesis fixation	APC-host bone fixation
1	Cemented	Uncemented
2	Cemented	Cemented
3	Cemented	Cemented
4	Cemented (16), uncemented (2)	Cemented(14), Uncemented(2), + plating(2)
5	Uncemented	Uncemented + plating
6	Cemented	Uncemented
7	Cemented	Cemented
8	Cemented	Uncemented (44), cemented (22)
9	Cemented	Uncemented (12), cemented (3)
10	NR	NR
11	Cemented	Cemented
12	Cemented	Uncemented
13	Cemented	Varied
14	Cemented	Uncemented
15	Cemented	Uncemented + plating
16	Cemented	Uncemented (13), cemented (2), + plating

**Table 4:** Table showing complications of prosthesis into allograft to form the allograft prosthesis composite (APC) and methods for securing APC to distal host femur. The total cohort included 498 patients with a mean follow up of 8.1 years (range 2 to 16.2 years). The pooled success rate was 81% (95% CI 77%–86%). Success rate: APC not revised. NR: not reported. Study numbers correlate with Table 1.

Study	*n*	Failed constructs	Success rate	Infection	Dislocation	Aseptic loosening or fracture
1	30	3	90%	1 (3.3%)	5 (16.7%)	4 (13.3%)
2	21	2	82%	0	0	6 (28.6%)
3	55	6	85%	2 (3.6%)	4 (7.3%)	5 (9.1%)
4	14	4	78%	3 (21.4%)	NR	1 (7.1%)
5	15	3	73%	1 (6.7%)	6 (40%)	2 (13.3%)
6	50	8	84%	2 (4%)	4 (8%)	7 (14.0%)
7	44	4	91%	1 (2.3%)	6 (13.6%)	3 (6.8%)
8	72	19	66%	5 (6.9%)	8 (11.1)	14 Loosening (4), resorption (3), nonunion (2), fracture (4), stem fracture (1)
9	15	2	87%	1 (6.7%)	1 (6.7%)	1 (6.7%)
10	73	13	82%	8 (10.9%)	0	11 (15.1%)
11	32	9	72%	4 (12.5%)	NR	5 (15.6%)
12	22	2	91%	1 (4.5%)	NR	1 (4.6%)
13	20	1	95%	1 (5.0%)	2 (10%)	0
14	25	2	92%	1 (4.0%)	NR	2 (8.0%)
15	37	10	73%	3 (8.1%)	NR	7 (18.9%)
16	15	5	67%	3 (20%)	1 (6.7)	4 (20.7%)
